# Arabidopsis antibody resources for functional studies in plants

**DOI:** 10.1038/s41598-020-78689-1

**Published:** 2020-12-15

**Authors:** Jaesung Oh, Michael Wilson, Kristine Hill, Nicola Leftley, Charlie Hodgman, Malcolm J. Bennett, Ranjan Swarup

**Affiliations:** 1 School of Biosciences and Centre for Plant Integrative Biology, University of Nottingham, Nottingham, UK; 2 Present Address: Plasma Technology Research Center, National Fusion Research Institute, Gunsan, Jeollabuk-do 573-540 Republic of Korea

**Keywords:** Cell biology, Immunology, Molecular biology, Plant sciences

## Abstract

Here we report creation of a unique and a very valuable resource for Plant Scientific community worldwide. In this era of post-genomics and modelling of multi-cellular systems using an integrative systems biology approach, better understanding of protein localization at sub-cellular, cellular and tissue levels is likely to result in better understanding of their function and role in cell and tissue dynamics, protein–protein interactions and protein regulatory networks. We have raised 94 antibodies against key Arabidopsis root proteins, using either small peptides or recombinant proteins. The success rate with the peptide antibodies was very low. We show that affinity purification of antibodies massively improved the detection rate. Of 70 protein antibodies, 38 (55%) antibodies could detect a signal with high confidence and 22 of these antibodies are of immunocytochemistry grade. The targets include key proteins involved in hormone synthesis, transport and perception, membrane trafficking related proteins and several sub cellular marker proteins. These antibodies are available from the Nottingham Arabidopsis Stock Centre.

## Introduction

The availability of full genome sequences and detailed RNA and protein expression databases has greatly increased our understanding of biological processes and functions at cellular, tissue and organ levels and has been extremely crucial in modelling multi-cellular systems using an integrative systems biology approach^1–3^. However, these models are based very often on assumptions regarding localization and sub cellular localization of key proteins, and refinement of these models will come from better understanding of their actual localization. This is likely to result in deeper understanding of both their function and their role in cell and tissue dynamics, including elucidating protein regulatory networks.

Many bioinformatics approaches have been developed to infer localization of a protein in a given cellular compartment^4–8^. Despite these methods, the prediction does not always fully match the experimental data^9^, so localization of the proteins in vivo must be confirmed. Biochemical and proteomic approaches to investigate protein localization by subcellular fractionation have also been proposed^10^ but cross-contamination very often is unavoidable. Localization of proteins by isotope tagging (LOPIT)^11,12^ attempts to address these issues, but these methods are based on statistical probability and still require confirmation of localization by alternative approaches. Proximity tagging methods^13^ identify proteins associated with a given cellular compartments but are not ideal for proteins localised in more than one compartment.

The two most popular methods for investigating localization are use of antibodies^14–16^ or protein fusions with fluorescent tags^17–19^. Antibodies are extremely powerful tools for protein localization studies and are widely used for a variety of other applications including western immunodetection, affinity purification, pull downs, chromatin immunoprecipitation (ChIP), ChIP-Chip, ChIP-Seq, enzyme-linked immunosorbent assays (ELISA) and fractionation studies. Alternative methods such as fusing small epitope tags (such as HA or FLAG) or fluorescent proteins (such as GFP or RFP) with the protein of interest are not ideal for a number of reasons: (a) they require the creation of transgenic organisms and thus may not truly represent endogenous protein levels because of the random nature of the integration of the transgene in the genome (position effect), (b) protein function may be affected by fusion to the tag, (c) sub-cellular protein localization may be affected due to the artificial nature of the fusion protein, and (d) protein abundance can be relatively hard to determine in their mis-sense mutants (because the wild type protein will rescue the mutant phenotype). Besides, investigating protein function in mutant backgrounds can be labour intensive and time consuming, as it will require crossing transgenic lines into those backgrounds.

Despite the usefulness and importance of antibodies, very often the availability of good quality antisera can be a limiting factor as they are time consuming and costly to produce. The Centre for Plant Integrative Biology (CPIB) has a big focus on root-related research (https://pubmed.ncbi.nlm.nih.gov/?term=centre%20for%20plant%20integrative%20biology%5BAffiliation%5D&sort=&pos=2) including the major aim of creating an atlas of key root proteins in the model plant *Arabidopsis*. Better understanding of expression, abundance and sub cellular localization of key root proteins in various mutant backgrounds, conditions and treatments will contribute towards a holistic understanding of their role in root development.

Here we summarise the results of the CPIB antibody project. We have raised 94 antibodies using either small peptides (up to 15 amino acids) or recombinant proteins using a simple pipeline. We compare the quality of the antibodies raised using these two approaches and show that many of the recombinant protein antibodies are able to detect correct target proteins. Thus, CPIB antibody resource is an extremely valuable communal resource for plant scientific community worldwide.

## Results and discussion

### Antibody pipeline

**Figure 1:**
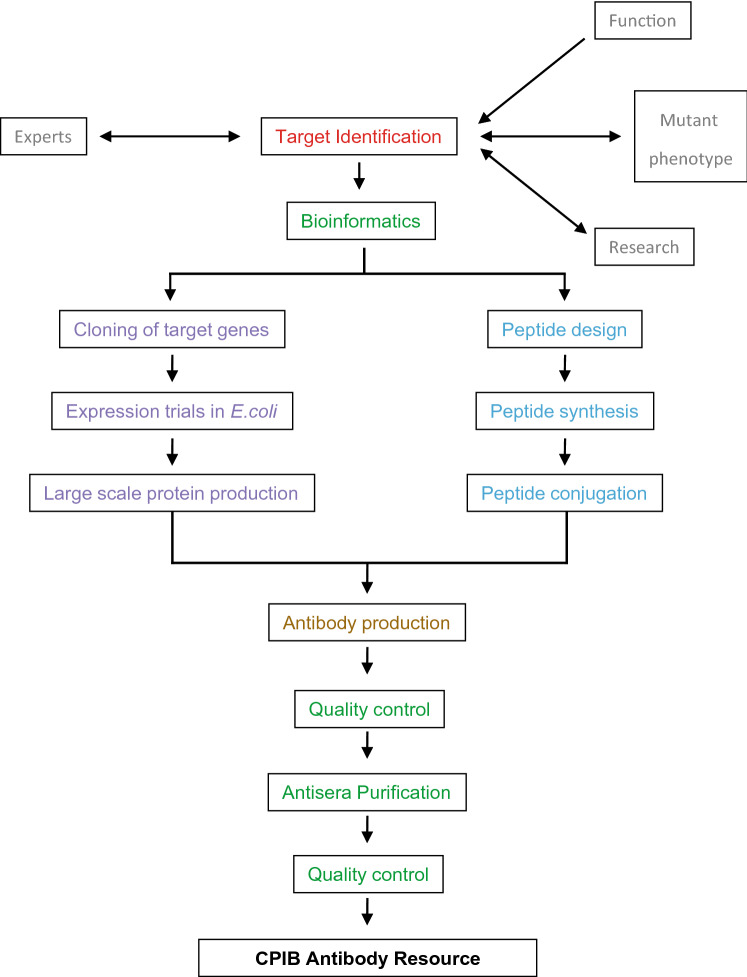
CPIB antibody pipeline. Targets for antibody production were identified and highly antigenic regions were determined by bioinformatics analysis. The proteins were then expressed in *E. coli*, purified by affinity chromatography and used for immunisation. Antibodies were then checked by dot blots, Westerns and in situ immuno-localisation and affinity purified if necessary.

The overall pipeline of antibody production is summarized in Fig. 1. It involved target selection, bioinformatic analysis of the target protein, identification of the antigenic regions within the protein, and probability analysis of chances of cross reactivity of the antigenic regions against non-target proteins. This was followed by cloning of the target region, antibody production and purification, quality control and validation.

### Target selection

The key root–protein targets were selected based on their role in root development, as judged by either root related developmental phenotype(s) or the importance of a given pathway in root development. The emphasis has been on plant hormones due to their importance in regulating several aspects of plant growth and development, including primary and lateral root development. Thus, some targets included proteins involved in plant hormone biosynthesis, transport and signaling. In addition, we have also raised antibodies against key cell-wall and cytoskeleton-related proteins, and 4 popular subcellular marker proteins BIP (endoplasmic reticulum), γ-cop (golgi), PM-ATPase (plasma membrane), and MDH (plastid) to facilitate co-localization studies. A complete list of all the target proteins used for antibody production can be seen in Table 1 and Supplementary Tables S1 and S2.

**Table 1:** List of successful antibodies raised in CPIB antibody project.

Name^Reference^	AGI code	Animal	Affinity purification	In situ IL	Protein mass (Kd)	Band detected in Western^a^
ACO2^30^	At1g62380	Sheep	Yes	Positive	36.2	OK
AXR1^31^	At1g05180	Sheep	Yes	Positive	60	~ 72, 55, 43, 10
AXR4^9^	At1g54990	Sheep	Yes	Positive	52.4	OK
AtBAP31^32^	At5g42570	Sheep	Yes	Positive	24.6	OK
BIM1^33^	At5g08130	Sheep	Yes	Positive	45.5	OK
BIP1^34^	At5g28540	Rabbit	Yes	Positive	73.6	OK
BRI1^35^	At4g39400	Sheep	Yes	Negative	130.5	~ 140,55,43
Catalase2^36^ (G)	At4g35090	Sheep	Yes	Positive	56.9	OK
AtCOB^37^	At5g60920	Sheep	Yes	Negative	51.2	OK and a few faint bands
γ-COP^38^	At4g34450	Rabbit	Yes	Positive	98.5	OK
GA3^39^	At5g25900	Sheep	Yes	Positive	58.2	~ 45, 50, 72, 130
GAI^40^	At1g14920	Sheep	Yes	Negative	58.9	OK
GNOM^41^	At1g13980	Sheep	Yes	Positive	162.6	OK
AtMDH^42^	At3g47520	Rabbit	Yes	Positive	42.4	OK
^b^LAX2^16^	At2g21050	Rabbit	Yes	Positive	54.6	NT
PIN1^43^	At1g73590	Sheep	No	Positive	67	~ 95, 120 and larger
PIN1^43^	At1g73590	rabbit	No	Positive	67	NT
PIN2^44^	At5g57090	Sheep	No	Positive	69.3	~ 95 kDa
PIN2^44^	At5g57090	rabbit	No	Positive	69.3	NT
PIN3^45^	At1g70940	Sheep	No	Positive	69.5	No band
PIN3^45^	At1g70940	rabbit	No	Positive	69.5	NT
PIN4^46^	At2g01420	Sheep	No	Positive	66.7	~ 84 kDa
PIN6^47^	At1g77110	Sheep	Yes	Positive	62	~ 115,30 k
PIN6^47^	At1g77110	rabbit	No	Positive	62	NT
PIN7^48^	At1g23080	Sheep	Yes	Positive	67.6	~ 62 kDa
PM-ATPase^49^	At2g18960	Rabbit	No	Positive	104.2	OK
RCN1^50^	At1g25490	Sheep	Yes	Negative	65.5	OK
RGA^51^	At2g01570	Sheep	Yes	Negative	64	OK
RSW1/CesA6/IRX2^52^	At5g64740	Sheep	Yes	Negative	122.5	OK?
RSW3^53^	At5g63840	Sheep	Yes	Negative	104.3	OK
SHR^54^	At4g37650	Sheep	Yes	Positive	59.5	~ 75 kDa
SLY1^55^	At4g24210	Sheep	Yes	Positive	17.5	> 130, 95
AtSYP21/AtPEP12^56^	At5g16830	Rabbit	Yes	Positive	31.1	OK and ~ 150 faint
AtSYP41/TLG^57^	At5g26980	Rabbit	Yes	Positive	36.1	OK
TIR1^58,59^	At3g62980	Sheep	Yes	Negative	66.8	OK
TWD^60^	At3g21640	Sheep	Yes	Negative	41.8	OK
V Invertase^61^	At1g12240	Sheep	Yes	Positive	73.8	~ 50, 30
WXR3y^62^	At3g45890	Sheep	Yes	Positive	66.4	~ 50, 45, 43

^a^Single correct size bands are indicated as ‘OK’. Where bands do not match the correct size or there are multiple bands, approximate band sizes (kDa) are indicated. *NT* not tested.

^b^Peptide antibody.

### Peptide v native or recombinant protein approach

There are two common approaches for antibody production, where animals are immunised against: (1) complete native protein or some parts thereof, or (2) small approximately 12–15 amino acid synthetic peptide (conjugated to an inert carrier protein)^20^. In addition, in a slight variation of the latter approach, short (3–5 amino acids) C-terminal-peptides have also been used successfully^21,22^. Despite their small size, Edwards et al*.*^21^ discovered that antibodies raised against these peptides were highly specific to their target protein and did not cross react with the similar internal sequences, suggesting that C terminal peptides may have a specific structure that can be exploited for antibody production.

The advantages of the peptide approach are that it is simple, convenient, and less likely to show non-specific cross reactivity. In comparison, the native or recombinant protein approach is more time consuming but increases the chances of a good immune response, due to the increased diversity and number of available epitopes.

### Peptide antibodies

**Figure 2:**
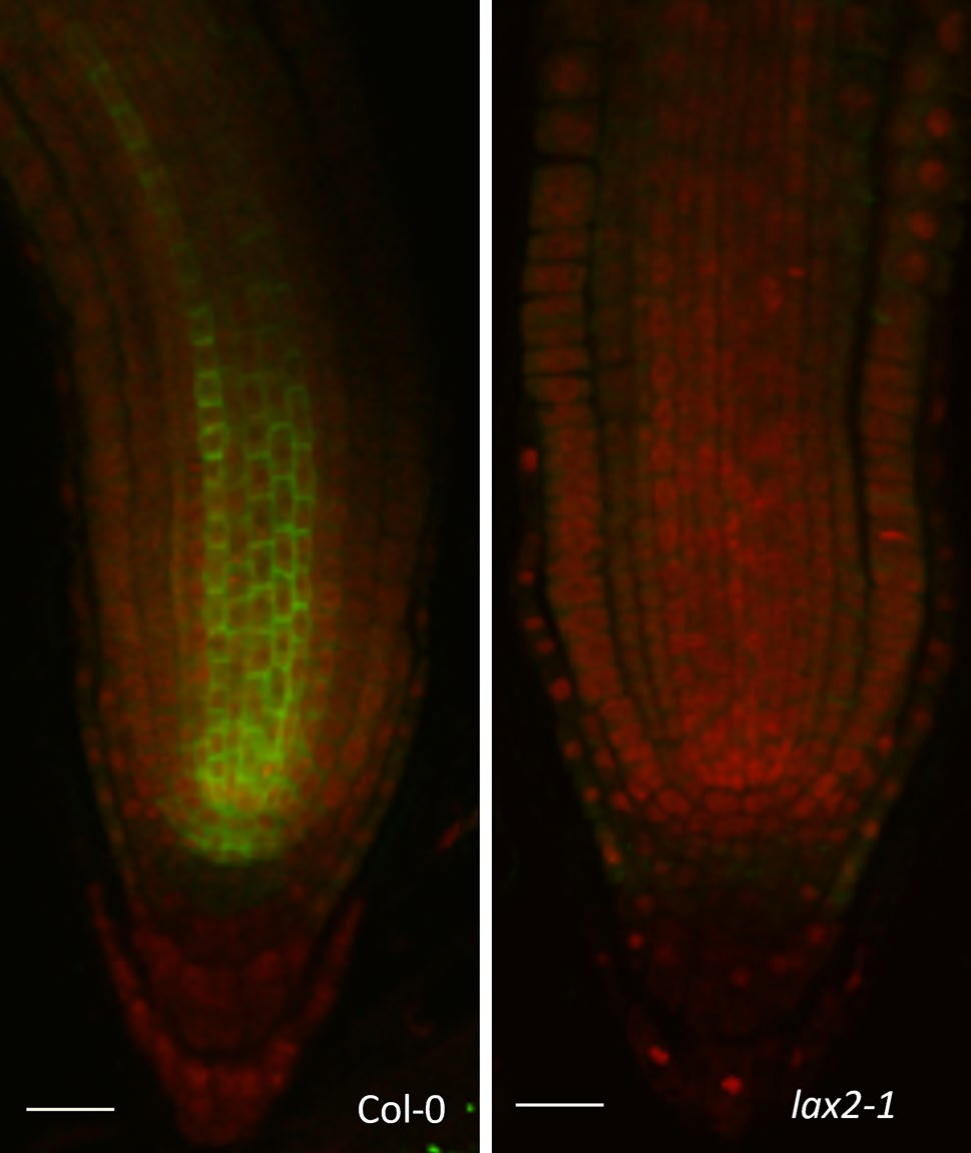
C-terminal antipeptide LAX2 antibody detects strong signal in wildtype Columbia (but not lax2 mutant) roots upon immunolocalization. Affinity purified LAX2 anti peptide antibody was used for in situ immunodetection of LAX2 (green) in 4-day old wild type Columbia or *lax2* mutant roots. Primary and Alexafluor 488 coupled secondary antibodies were used at 1:200 dilutions. Seedlings were counter stained using propidium iodide (red). Scale bar 20 μm.

Because of its simplicity and less chance of non-specific cross reactivity, both standard peptide and short C terminal peptide approaches were tried initially. Surprisingly, this did not work very well in our hands with a very poor success rate. Even after affinity purification against the peptide, the detection rate remained very low and only one out of 24 antibodies worked satisfactorily (Supplementary Table S1). The only antiserum that worked well was affinity purified LAX2 (LIKE AUX1-2). It appears to be very specific as it detected a strong signal in the root apex in wild type Columbia roots but not in null *lax2* mutants (Fig. 2).

For the 23 remaining antibodies, it is difficult to pinpoint why the success rate was so low, but one main reason could be the epitope prediction. The prediction methods identify individual stretches of amino acids (continuous epitopes), whereas epitopes are very often discontinuous, involving distant subsequences brought together by the protein’s tertiary structures^23^. However, prediction methods for the latter are not well developed and have met with little success. Also, a synthetic continuous (or even discontinuous epitope) peptide may still not fold correctly and hence not generate antibodies that recognize the native protein structure^23^.

Because of the low success rate of anti-peptide antibodies (from three different companies), this approach was abandoned, and efforts were turned to the recombinant-protein approach.

### Recombinant-protein antibodies

**Figure 3:**
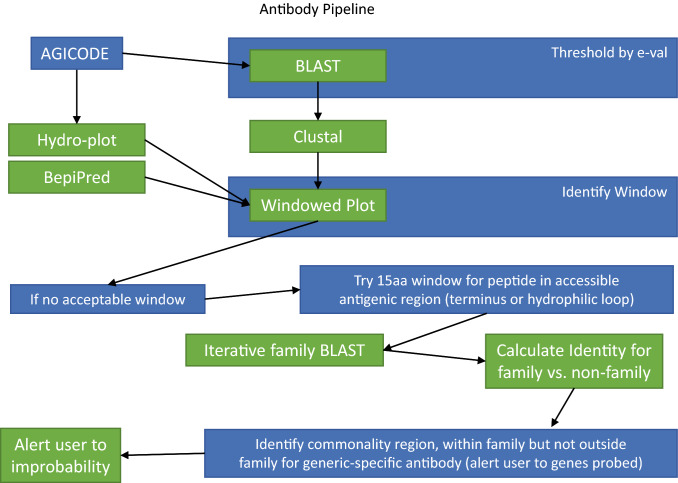
Bioinformatics approach used for identification of protein segments used for antibody production.

70 antibodies were raised against *Arabidopsis* root proteins using the recombinant protein approach (Supplementary Table S2). Bioinformatic analysis was used to identify potential antigenic regions and then the largest antigenic subsequence was checked for potential cross-reactivity by database searches using blastX^24^ (Fig. 3). A cut off of 40% similarity score (at amino acid level) was used as a guide to accept a given antigenic region for antibody production. In cases where blast results exceeded the cut off, we either chose another antigenic region or used a sliding window to obtain a smaller region that showed less than 40% sequence similarity. However, in cases of multi-gene families where it was not possible to obtain a reasonably large (~ 100 amino-acid) unique sequence, a more generic family-specific antibody was raised. Where possible, antibody cross reactivity was tested in the corresponding mutant backgrounds by either western immune detection and/or by in situ localization.

**Figure 4:**
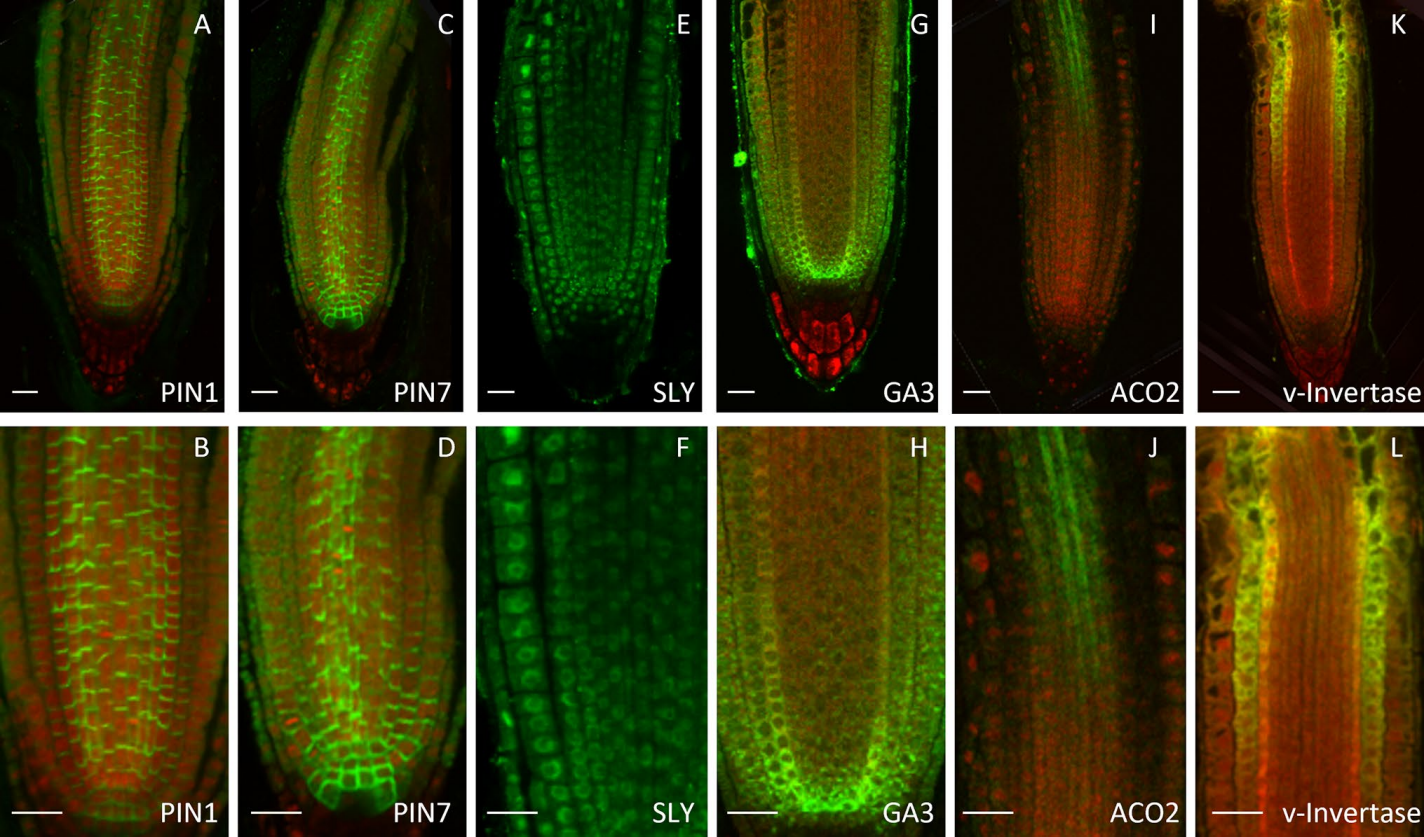
In situ immunodetection of root proteins. Crude (**A**–**D**) or affinity purified (**E**–**L**) antisera were used for in situ immunodetection of the target proteins (green) in 4-day old Columbia roots. Primary and Alexafluor 488 coupled secondary antibodies were used at 1:200 dilutions. Seedlings were counter stained using propidium iodide (red). Scale bar 20 μm.

**Figure 5:**
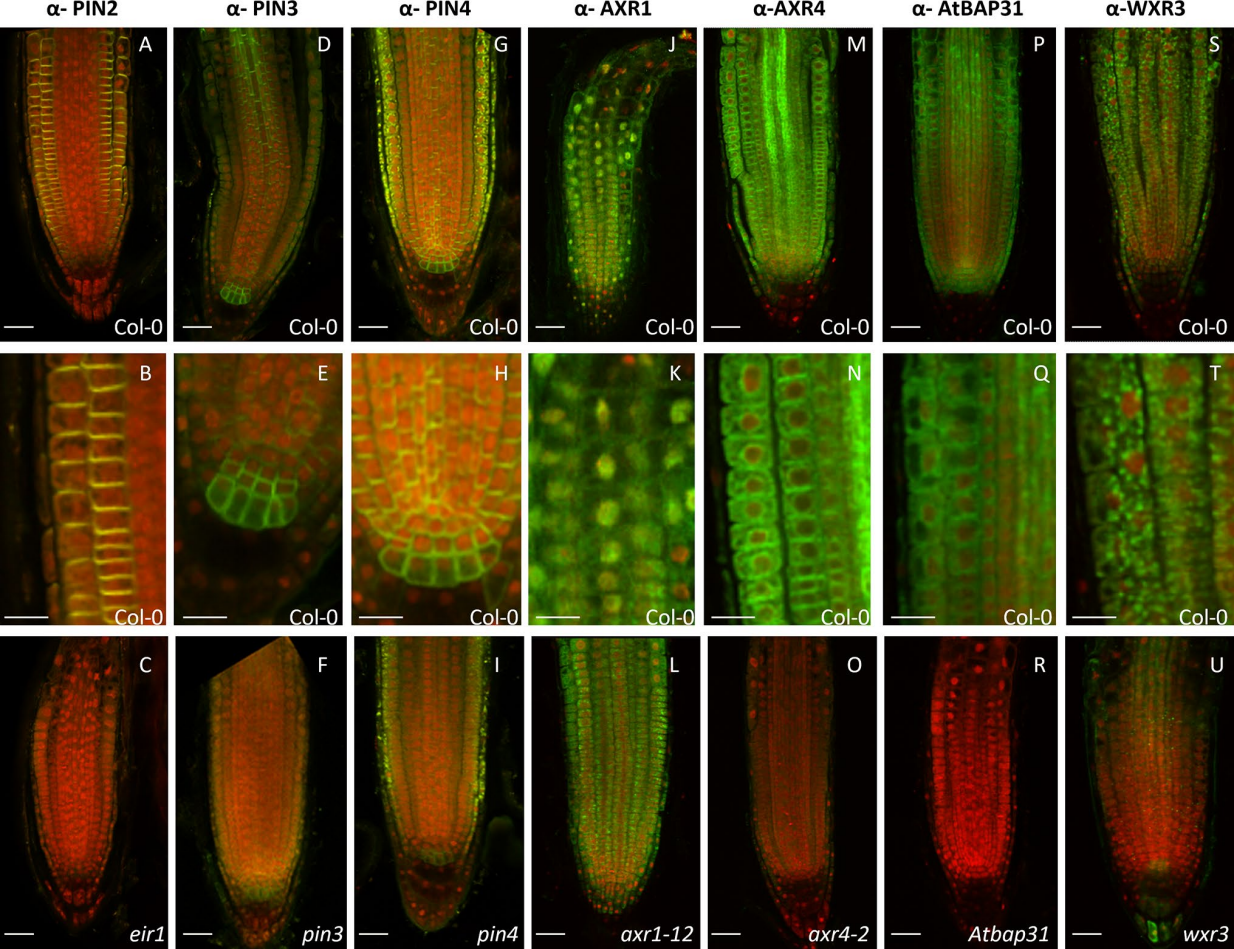
Typically, CPIB antibodies do not show non-specific cross reactivity upon in situ immunolocalization. Crude (**A**–**I**) or affinity purified (**J**–**U**) antisera were used for in situ immunodetection of the target proteins (green) in 4 day old wild type Columbia (**A**,**B**,**D**,**E**,**G**,**H**,**J**,**K**,**M**,**N**,**P**,**Q**,**S**,**T**) or respective mutant roots (**C**,**F**,**I**,**L**,**O**,**R**,**U**). Primary and Alexafluor 488 coupled secondary antibodies were used at 1:200 dilutions. Seedlings were counter stained using propidium iodide (red). Middle panel (**B**,**E**,**H**,**K**,**N**,**Q**,**T**) is close ups of the expression domain. Scale bar top and bottom panels 20 μm, middle panel 10 μm.

**Figure 6:**
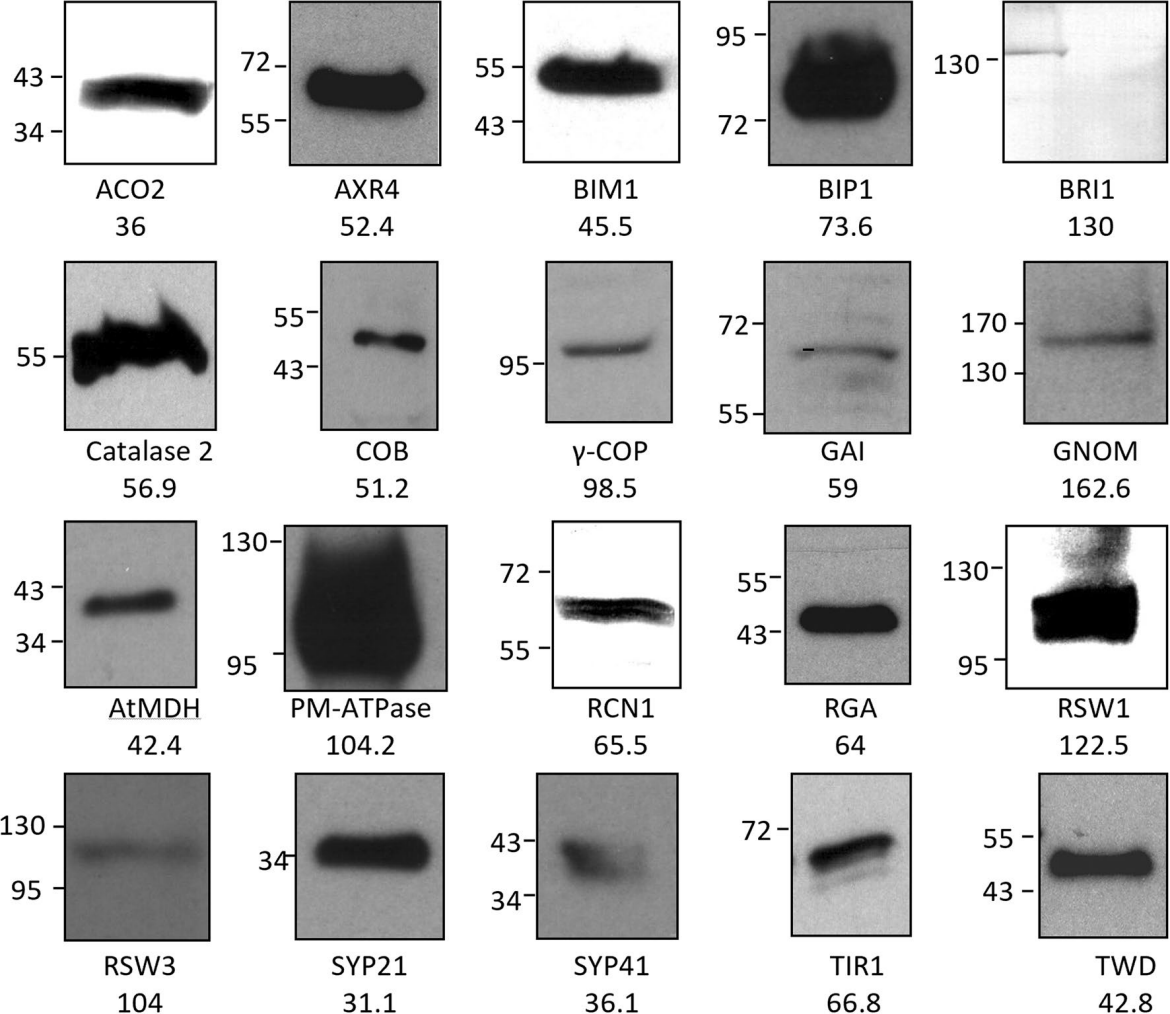
Typically, CPIB antibodies show single correct size band upon western immunodetection. Twenty-five microgram of *Arabidopsis* total proteins were separated by SDS-PAGE and transferred to PVDF membranes. The blots were then used for Western immunodetection of target proteins using affinity-purified primary antibodies and HRP conjugated secondary antibodies. Marker sizes are indicated on the left of the bands whereas expected band sizes are indicated below the images.

**Figure 7:**
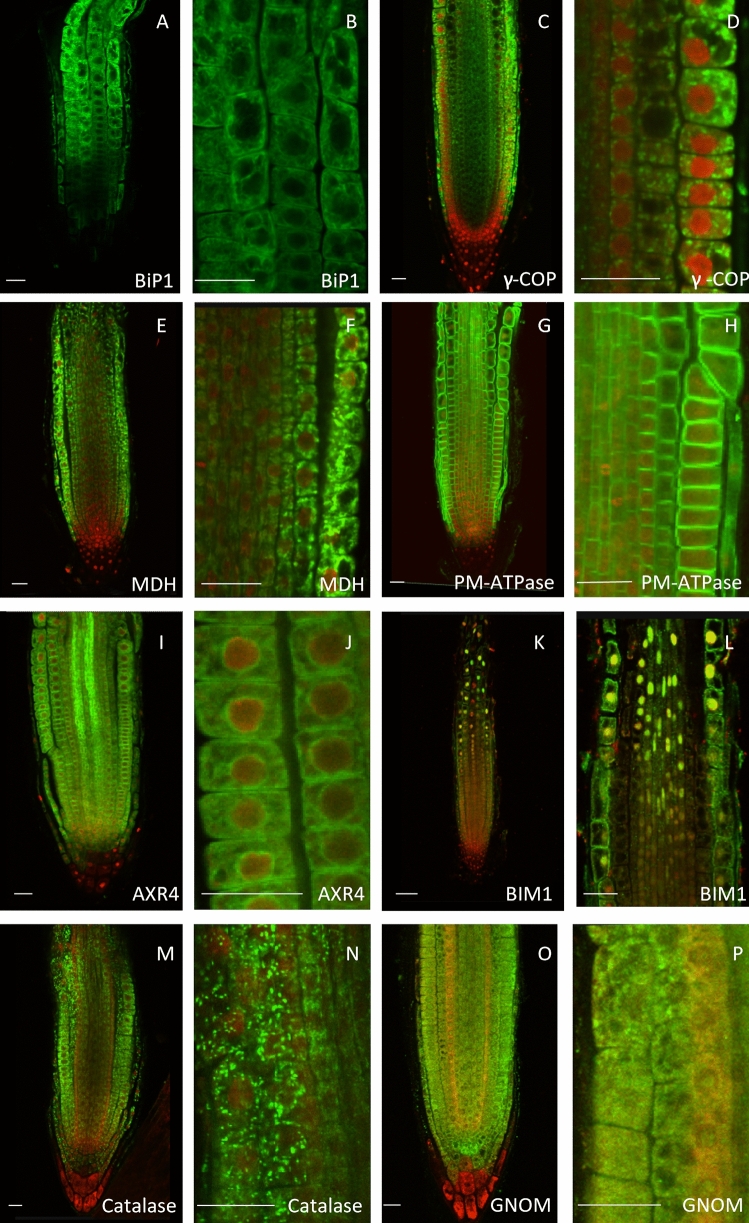
CPIB antibody project has raised antibodies against several popular sub-cellular markers: BiP (ER), γ-cop (golgi), PM-ATPase (PM) and malate dehydrogenase (plastid). Antibodies were raised against popular sub cellular marker proteins (**A**–**H**) and other root proteins (AXR4 (ER), BIM1 (nucleus), catalase (peroxisome) and GNOM (endosome) that potentially can be used as subcellular markers (**I**–**P**). The antibodies were used for immunodetection of the targets (green) in 4-day old wild type Columbia roots. Primary and Alexafluor 488 coupled secondary antibodies were used at 1:200 dilutions. Seedlings were counter stained using propidium iodide (red). Scale bar 20 μm.

Initial quality control using dot blots against the recombinant protein revealed that most crude antisera could detect the target proteins in the picogram range, indicating a good titre (Supplementary Fig. S2). However, most of the crude antibodies did not show any signal when tested by in situ immunolocalization, the exceptions were PIN1, PIN2, PIN3, PIN4, PIN7 and PM-ATPase. Generic purification methods such as Caprylic acid precipitation^25^, Protein A or Protein G purification^26^ and signal amplification methods^27^ did not improve the detection rate. Whereas affinity purification with the purified recombinant protein (Supplementary Fig. S3) resulted in significant improvement in detection rate: 38 (55%) antibodies could detect a signal with high confidence either by in situ immunolocalization (22 out of 38) or Westerns (20 out of 32 tested) or both (Figs. 4, 5, 6, 7).

A complete list of useful antibodies is given in Table 1. As can be seen, successful targets include several key proteins involved in hormone synthesis, transport and perception (LAX2, PIN proteins, AXR1, TIR1, GA3, GAI, RGA, GID, SLY1, ACO1), membrane trafficking related proteins (AXR4, GNOM, AtSYP21, AtSYP41) and other important root proteins including SHR, RSW1, RSW3 and WXR3. In several cases, the validity of the signal was checked against the respective mutant backgrounds by in situ immunolocalization (Fig. 5) or by detecting a single band, usually of the expected size, on Western blots (Fig. 6 and Supplementary Fig. S4). As evident in Fig. 5 (and also Fig. 2; Supplementary Fig. S3), all the antibodies that were checked against their mutant background for cross reactivity by in situ immunolocalization gave no detectable signal in the mutants except for anti-PIN3 where a faint signal was detected. This suggests that the bioinformatics approach that we used for our antibody pipeline is a robust approach and can be used as a guide for other antibody projects.

Despite significant improvement in detection rates, several antisera still could not detect a signal by in situ immunolocalization. In some cases, this could be attributed to poor immune response in animals as the quality of the affinity purification was not very good, resulting in low levels of the IgG. But in other cases, despite good quality of affinity purification, no signal was detected. We cannot rule out the possibility that the target proteins are low abundant and hence are below the limits of our in-situ detection. Similarly, for westerns, in some cases, we did not detect a correct size band (Fig. 6; Supplementary Fig. S4). It is likely that some of this can be attributed to degradation or post-translational modifications or for some membrane proteins could be attributed to poor migration on the gel due to hydrophobic nature of these proteins. For most other proteins, we did detect a single correct size band (Fig. 6; Supplementary Fig. S4). Like in situ immunolocalisation (Figs. 2, 5, Supplementary Fig. S3), validation for a few antibodies (AXR4, ACO2, AtBAP31 and ARF19) have also been validated by westerns against their respective mutant backgrounds (data not shown). We envisage that with the help of the community, as more researchers use this resource, other antibodies will also be validated, and this information will be constantly reviewed and updated on Arabidopsis Stock Centre pages.

Sub-cellular markers are an extremely useful tool and are used for several applications including colocalization or fractionation studies^3,9,18^. As part of the CPIB antibody project, we also have raised antibodies against 4 popular sub-cellular markers (BiP, γ-cop, PM-ATPase and MDH), and also α-AXR4 (endoplasmic reticulum), α-AtBIM1/AtbHLH046 (nucleus), α-CATALASE (peroxisome) and α-GNOM (endosome) because of their high expression in almost all cell files in the roots (Fig. 7).

In conclusion, we have raised 94 antibodies against key root-proteins using either small peptides (up to 15 amino acids) or recombinant proteins. Thirty-eight of these antibodies appear to be of good quality and 22 are of immunocytochemistry grade. CPIB antibodies form an extremely valuable communal resource for plant scientific community worldwide and will be available from Nottingham Arabidopsis Stock Centre (NASC).

## Materials and methods

### Cloning, expression and purification

The target sequences for antibody production were chosen based on antigenicity plots (DNASTAR) and blastX^24^. The target sequences were PCR amplified from a 5-day-old root-cDNA library, using gene specific primers and cloned into linearized pENTR/Directional-TOPO vector (Thermofisher Scientific) as per manufacturer’s instructions. Positive clones were identified by colony PCR and further confirmed by sequencing. These entry vectors were then recombined into the gateway destination vector pDEST17 to create N terminal translation fusions with a 6 × Histidine tag (6xHis) as per manufacturer’s instructions. Positive colonies were identified by colony PCR and plasmid DNA was further validated by PCR and restriction digestion. Finally, these recombinant plasmids were transformed into *E. coli* expression strains Rosetta or BL21-AI (Thermofisher Scientific).

Three or four colonies were initially tested in a small-scale expression trial. For BL21-AI, small-scale induction trials were run as per manufacturer’s instructions. In short, overnight cultures were used to inoculate (1:100 dilution) fresh growth media (Luria broth) containing appropriate antibiotics and allowed them to grow to an OD_600_ of about 0.4. The expression of the target protein was induced by Arabinose (0.2%) and 0.5 ml samples were withdrawn at 0, 2, 4 and 24 h after induction. Samples were centrifuged and the pellet was heated in sample buffer at 95C for five minutes and separated by SDS-PAGE. For Rosetta, the auto-induction method^28^ was used and pelleted cells were heated in sample buffer as above and subjected to SDS-PAGE.

For large scale protein production, single colonies were grown for 16 h at 37 °C in 3 ml LB. 1 ml of this culture was then used to inoculate 400 ml of auto induction medium and grown for 16 h at 37 °C. The cells were harvested at 6000 rpm for 5 min and were resuspended in 10 ml of Binding Buffer (8 M Urea/40 mM Sodium Phosphate Buffer, pH7.4/0.3 M NaCl/20 mM Imidazole), then solubilized for 1 h at room temperature with gentle shaking. The lysate was sonicated for 30 min in ice using a water-bath sonicator and centrifuged at 8000 rpm for 45 min. The supernatant was then passed through 0.45 µm sterile filter and purified straightaway using the ÄKTAxpress protein-purification system (GE Healthcare, UK).

A two-column purification strategy was used that combined affinity purification (HisTrap nickel columns-GE Healthcare, UK) with desalting. A typical purification protocol involved equilibration of the HisTrap column with 2 column volumes of binding buffer; passage of the cleared lysate through the equilibrated column; washing of the column with five column volumes of binding solution; followed by elution of the recombinant His-tagged protein using a 20–500 mM continuous imidazole gradient. The machine was programmed to pass the largest peak on to a 2 × Sephadex G25 column assembly and 2 ml fractions were collected through a built-in fraction collector. Peak protein fractions were then used for protein assay by the Bradford method^29^, and the extent of purification was subsequently checked by SDS-PAGE.

### Antibody production

One milligram of protein was sent to Scottish National Blood Transfusion Services (Edinburgh, UK) for immunization, which consisted of a primary injection and two booster injections 4 weeks apart, with test bleeds were taken at 4, 8 and 12 weeks after the initial injection.

### Affinity purification of antibody

One ml of Sulfo-Link Coupling Resin (Thermo Scientific, UK) was placed into a disposable 5 ml polypropylene column (Thermo Scientific, UK) and equilibrated with 5 ml of coupling buffer (50 mM Tris–HCl, pH8.5/5 mM EDTA). Five hundred micrograms of the recombinant protein were added to the column and incubated with rotation for 30 min at room temperature. The column was placed upright for 30 min without mixing, followed by washing with 3 ml of coupling buffer. Two mls of 50 mM of Cysteine in coupling buffer was added to the column and rotated for 15 min at room temperature, followed by washing with 6 ml of 1 M NaCl. The column was washed with 20 ml of 10 mM Tris–HCl, pH7.5/0.5 M NaCl, 10 ml of 100 mM Glycine (pH2.5), and 20 ml of 10 mM Tris–HCl, pH7.5. Fifty mls of 1 M Tris–HCl, pH7.5 were added to 5 ml of crude antiserum and filtered through with 0.45 µm filter. The buffered antiserum was added to the column and incubated with rotation overnight at 4 °C. On the next day, the antiserum was allowed to drain out, and flow-through was passed over the column twice at room temperature. The column was washed with 20 ml Tris–HCl, pH7.5 and 10 ml of 10 mM Tris–HCl (pH7.5)/0.5 M NaCl. Two-hundred and fifty mls of antibody were collected into 1.5 ml Eppendorf tubes containing 50 µl of 1 M Tris–HCl, pH8.0 with 2 ml of 100 mM Glycine, pH2.5. Each fraction was used for measurement of protein concentration and SDS-PAGE. Fractions were stored at − 80 °C.

### Dot blot

As a measure of the antibody titre, antisera were tested on dot blots. These were prepared by spotting 10 ng to 100 pg of expressed recombinant proteins on nitrocellulose or PVDF membranes which were used for western immunodetection. In short: blocking (5% Non-fat milk-1 h); primary antibody (1:200 dilution-1 h); washing (3 × 5 min in TBST (Tris buffered saline; 0.1% Tween20); secondary antibody (alkaline phosphatase conjugate—1:5000-1 h); washing ((3 × 5 min in TBST) and detection using NBT/BCIP substrate solution in 0.1 M Tris–HCl buffer pH 9.5 containing 0.1 M NaCl and 0.05 M MgCl_2._

### Plant protein extraction

Five to 7 day old Arabidopsis seedlings or 4 week old root cultures were ground using liquid nitrogen and homogenized in homogenization buffer (50 mM HEPES, pH7.5/0.5 M sucrose/0.1% sodium ascorbate/1 mM DTT/0.5% polyvinyl polypyrolidone, insoluble/protease inhibitor), centrifuged and the crude extracts were used for protein estimation by the Bradford method^29^. For membrane proteins, microsomal fractions were prepared as described previously^18^.

### Western immunodetection

Proteins (25 µg) were separated by SDS-PAGE and transferred to PVDF membrane using Trans-Blot Semi-Dry Electrophoretic Transfer Cell (Bio-Rad). These membranes were probed as described above for dot blots with some modification. Primary antibodies were normally used at a dilution of 1:200–1:5000 (37 °C 12–16 h) whereas secondary antibodies were routinely used at a dilution of 1:5000 (37 °C 2–3 h).

### In situ immunolocalization

This was carried out on three to 4-day old Arabidopsis roots as described previously^3,14,16^. Crude or affinity-purified antisera were used at 1:50–1:400 dilutions (37C 5 h) whereas secondary antibodies were used normally at 1:200 dilutions (37C 5 h). The images were captured using Leica SP2 confocal laser scanning microscope (Leica Microsystems UK Ltd).

### Ethical statements

As the animals were involved in the antibody production through several companies, we have enquired with the companies and we can confirm that (1) all experimental protocols were approved by a named institutional and/or licensing committee/s. (IACUC committee; PTU/BS has a project licence under the Animals (Scientific Procedures) Act 1986, which permits contract immunisation of animals and (2) all methods were carried out in accordance with relevant guidelines and regulations (USDA guidelines; PTU/SB work is carried out in compliance with the requirements of ISO9001).

## Supplementary information

Supplementary Information.

## Data Availability

All the antibodies will be available from Nottingham Arabidopsis Stock Centre (NASC).
