# Supplementary material for: Arabidopsis antibody resources for functional studies in plants
Source: Sci Rep. 2020 Dec 15;10:21945. doi: 10.1038/s41598-020-78689-1 (PMC7738516; doi:10.1038/s41598-020-78689-1)
Supplement: Supplementary file 1 — Supplementary Information. [file 41598_2020_78689_MOESM1_ESM.docx]

**Supplementary information**

**Arabidopsis antibody resources for functional studies in plants**

Jaesung Oh^1,2^, Michael Wilson^1^, Kristine Hill^1^, Nicola Leftley^1^, Charlie Hodgman^1^, Malcolm J Bennett^1^, Ranjan Swarup^1*^

^1^ School of Biosciences and Centre for Plant Integrative Biology, University of Nottingham, UK.

^2^ Current Address

Plasma Technology Research Center, National Fusion Research Institute

Gunsan-City, Jeollabuk-Do, 573-540 Republic of Korea (ohjs@nfri.re.kr)

* corresponding authors Ranjan Swarup (ranjan.swarup@nottingham.ac.uk)

**Supplementary Figure 1: Affinity purification of 6 x His tagged proteins**

His tagged target proteins (eg PLT, BIM1 and PMATPase) were expressed in *E. coli*. Crude bacterial lysates were affinity purified and desalted using HisTrap and HiTrap columns (GE Healthcare), respectively. Crude lysates and purified protein fractions and were then checked by SDS-Polyacrylamide gel electrophoresis.

PM ATPase

PLT

BIM1


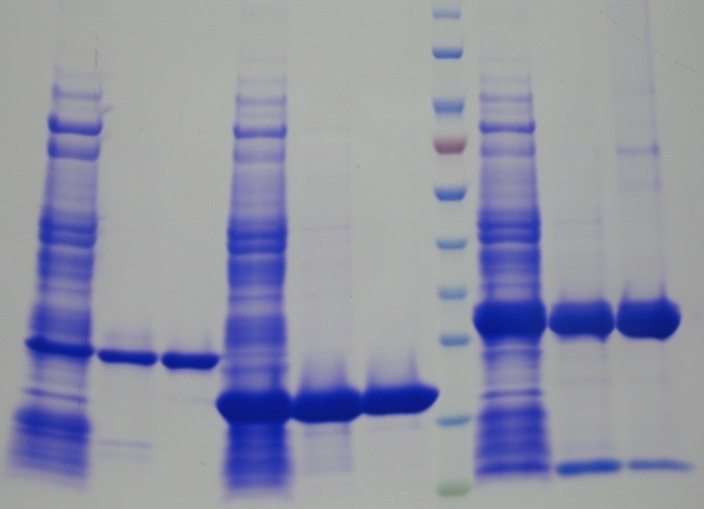


17

26

34

43

Crude

M

Purified

Crude

Purified

Crude

Purified

Kd

**Supplementary Figure 2: Immunodetection of target proteins by dot blots**

Nanogram and pictogram quantities of purified recombinant proteins were spotted on nylon membranes and were then used for immunodetection using crude or affinity-purified primary antibodies and alkaline phosphatase conjugated secondary antibodies.

Generic antibodies are indicated with an asterisk (*).


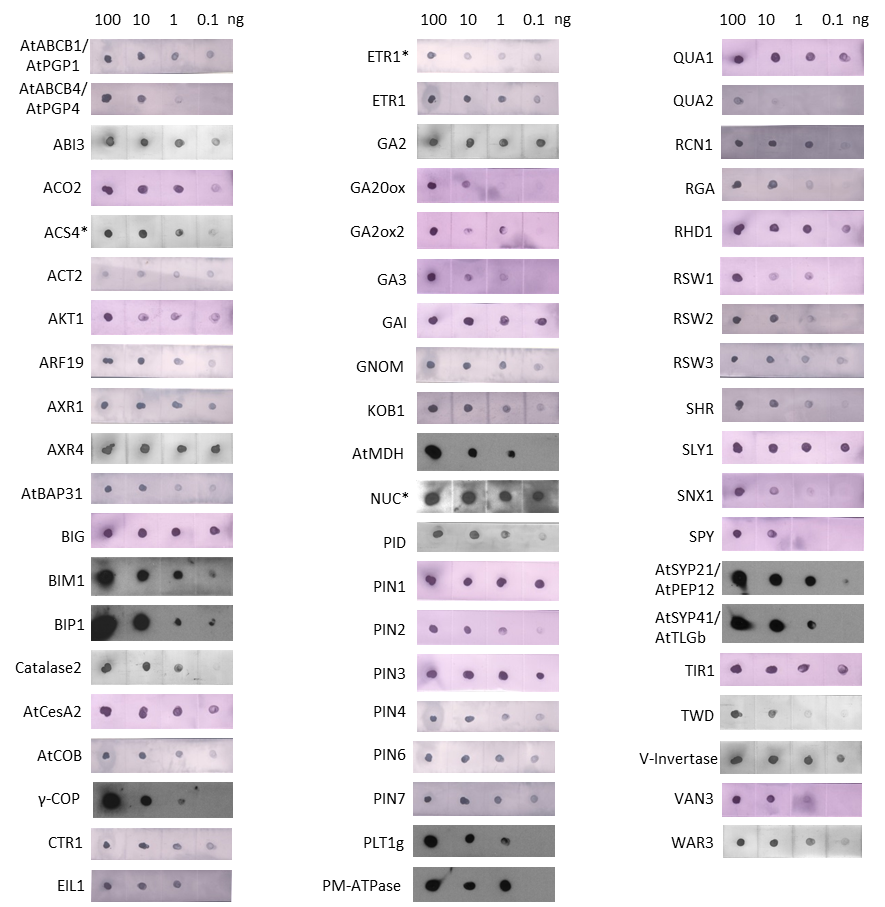


**Supplementary Figure 3: Affinity purification of crude antisera significantly improves detection rates**

1. *E. coli* expressed proteins were coupled to sulfo-link resins and used for affinity purification of crude anti sera. In this example, crude SHR antiserum was purified on a SHR coupled sulfo-link resin and fractions were checked by SDA-PAGE. IgG heavy and light chain bands are marked with black arrows.
2. SHR (green) was immunodetected in 4-day old Columbia or *shr101* roots using affinity purified SHR primary antibody (1:200 dilution) and Alexafluor 488 coupled anti sheep secondary antibody (1:200 dilution). Seedlings were counter stained using Propidium Iodide (red). Scale bar 10 μm.


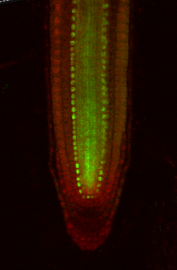

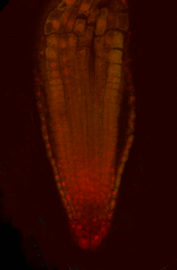


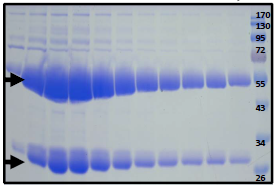


IgG

Heavy

Chain

IgG

Light

Chain

Fractions

**A**

55

34

26

72

Kd

130

**B**

*shr101*

Col-0

**Supplementary Figure 4: Typically, CPIB antibodies show single correct size band upon western immunodetection**

Typically, twenty-five microgram of *Arabidopsis* total proteins were separated by SDS-PAGE and transferred to PVDF membranes. The blots were then used for Western immunodetection of target proteins using affinity-purified primary antibodies and HRP conjugated secondary antibodies. Marker sizes (kd) are indicated on the left of the bands whereas expected band sizes are indicated below the pictures.


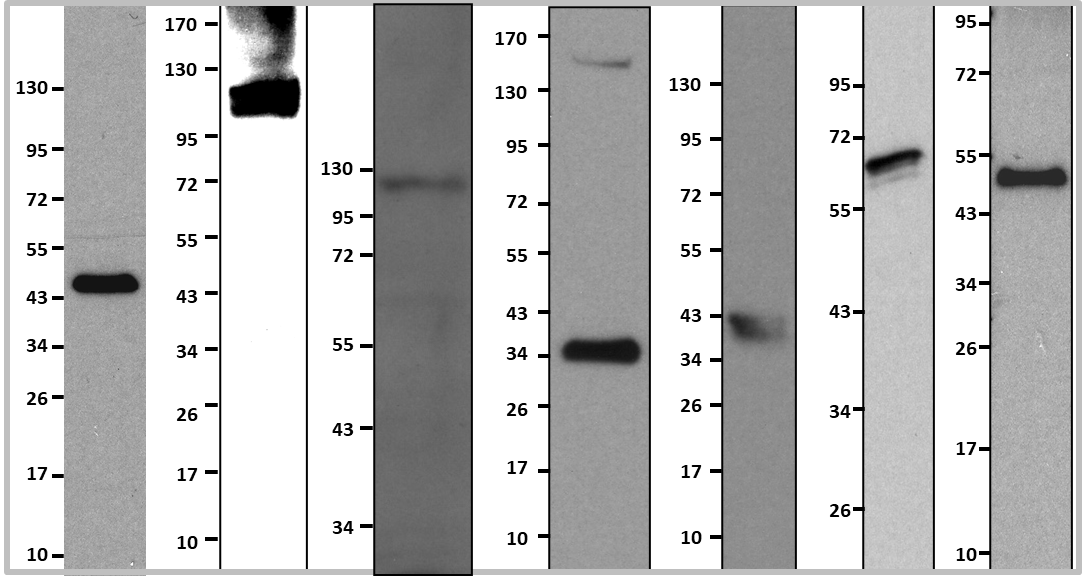

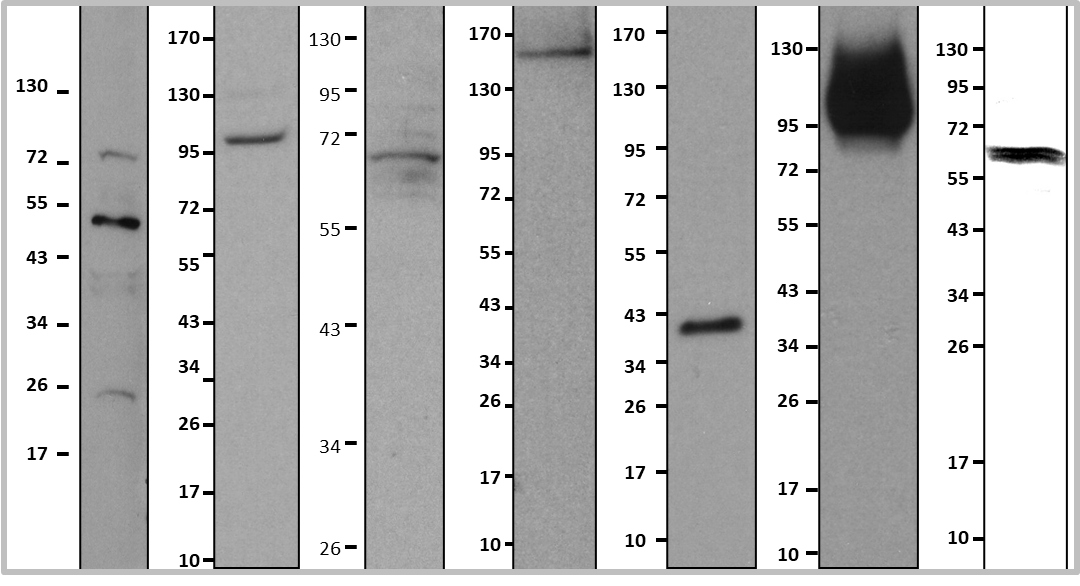

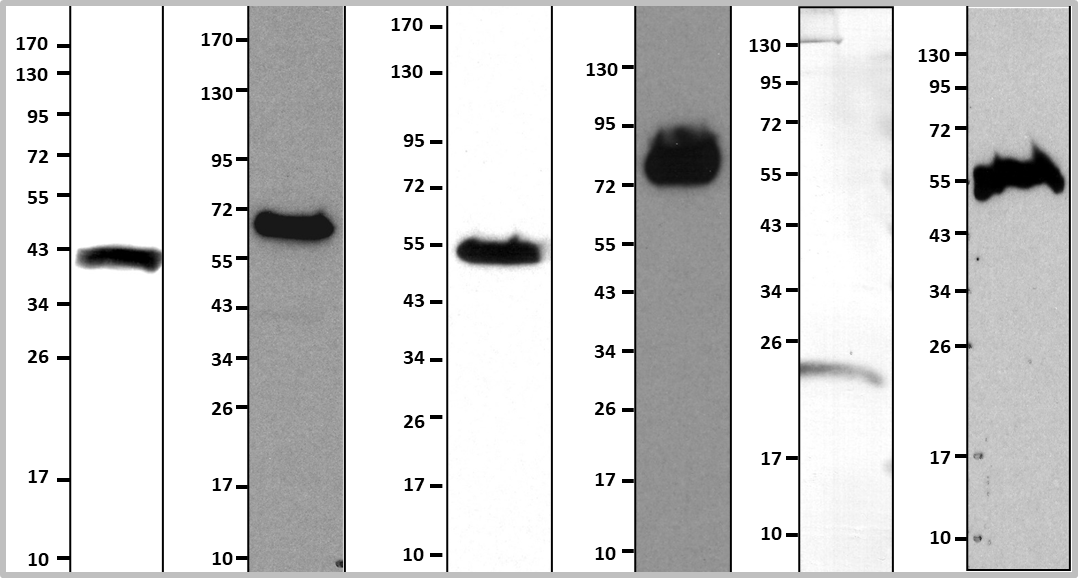


ACO2

36.2

α-gCatalase2

56.9

α-BRI1

130.5

α-BIM1

58.7

α-BIP1

73.6k

AXR4

52.4

COB

51.2

AtMDH

42.4

PM-ATPase

104.2

GNOM

162.6

γ-COP

98.5

RCN1

65.5

GAI

59

TWD

42.8

RGA

64

TIR1

66.8

RSW1

122.5

SYP41

36.1

SYP21

31.1

RSW3

104

**Supplementary Table 1: List of CPIB peptide antibodies**

| Gene | Name | Position | Sequence | Animal | Affinity Purified | Localisation |
| --- | --- | --- | --- | --- | --- | --- |
| At2g38120 | AUX1 | 22-33 | RTGKDNE(C)EHDGSTGS | Goat |  | Negative |
|  |  | 22-33 | RTGKDNE(C)EHDGSTGS | Rabbit | Yes | Negative |
|  |  | 22-32 | RTGKDNEEHDG(C) | Rabbit | Yes | Negative |
|  |  | 480-485  C terminal short | (C)ALHHRL | Goat |  | Negative |
| At1g73590 | PIN1 | 617-622 C terminal short | (C)YILLGL | Goat |  | Negative |
| At5g57090 | PIN2 | 455-468 | (C)GGKSPYMGKKGSDV | Goat |  | Negative |
| At4g37650 | SHR | 173-185 | SSPYGDTEQKLASC | Goat |  | Negative |
|  | SHR | 526-531 C terminal short | (C)SAWRPT | Goat |  | Negative |
| AT3G62980 | TIR1 | 575-588 | (C)NMDQDSTMRFSRQI | Rabbit | Yes | Negative |
| At4g03190 | AFB1 | 571-585 C terminal | (C)IHKNPENGVSHLAIK | Rabbit | Yes | Negative |
| At3g26810 | AFB2 | 543-550 | (C)ENGHEGRQ | Rabbit | Yes | Negative |
| At1g12820 | AFB3 | 346-355 | (C)VHGEEDNNAS | Rabbit | Yes | Negative |
| At5g01240 | LAX1 | 22-30 | RKVEDSAAE(C) | Rabbit | Yes | Negative |
|  |  | 474-488 C terminal | (C)KPPPAPIAAGAHHRR | Rabbit | Yes | Negative |
| At2g21050 | LAX2 | 471-483  C terminal | (C)PISHPHFNHTHGL | Rabbit | Yes | Positive |
| At2g21050 | LAX2 | 471-483 C terminal | (C)PISHPHFNHTHGL | Rabbit | Yes | Negative |
| AT1G77690 | LAX3 | 25-34 | SGNKKSSTKT(C) | Rabbit | Yes | Negative |
|  |  | 456-470 | (C)FGLFTK(C)YQ(C)PPHKP | Rabbit | Yes | Negative |
| At1g66340 | AtETR1 | 729-738 C terminal | (C)LEPRVLYEGM | Rabbit | Yes | Negative |
|  |  | 674-888 | (C)IHEKFTKQRHQRPLL | Rabbit | Yes | Negative |
| At3g23150 | AtETR2 | 623-633 | (C)HGSSESPAPDH | Rabbit | Yes | Negative |
| At2g40940 | AtERS1 | 600-613 C terminal | (C)LHLAAKSQTRPWNW | Rabbit | Yes | Negative |
| At1g04310 | AtERS2 | 633-645 C terminal | (C)ETFRTSTPPSTSH | Rabbit | Yes | Negative |
| At3g04580 | AtEIN4 | 754-766 C terminal | (C)ASELRRALQTASE | Rabbit | Yes | Negative |

**Supplementary Table 2: List of CPIB recombinant protein antibodies**

| **Gene** | AGI code | Animal | Antigenic Region | Quality of Affinity Purification | Immuno-localisation | Proteins Mass (kda) | Western* |
| --- | --- | --- | --- | --- | --- | --- | --- |
| **AtABCB1/AtPGP1** | At2g36910 | Sheep | 592-688 | Poor | Negative | 140.6 | No Band |
| **AtABCB4/AtPGP4** | At2g47000 | Sheep | 621-731 | Good | Negative | 139 | No Band |
| **AtABCB19/AtPGP19** | At3g28860 | Sheep | 607-674 | V Poor | Negative | 136.8 | No Band |
| **ABI3** | At3g24650 | Sheep | 251-470 | V Poor | Negative | 79.5 | NT |
| **ACO2** | At1g62380 | Sheep | 1-320 | V Good | Positive | 36.2 | OK |
| **ACS4 (Generic)** | At2g22810 | Sheep | 270-412 | Good(?) | ? | 53.8 | No Band |
| **ACT2** | At3g18780 | Sheep | 181-338 | V Poor | Negative | 41.9 | ~48k |
| **AKT1** | At2g26650 | Sheep | 498-750 | V Good | Negative | 97 | No Band |
| **ARF19** | At1g19220 | Sheep | 541-720 | V Good | Negative | 120.6 | OK nuclear prep, 60 |
| **AXR1** | At1g05180 | Sheep | 144-425 | V Good | Positive | 60 | ~72, 55, 43, 10 |
| **AXR4** | At1g54990 | Sheep | 301-473 | V Good | Positive | 52.4 | OK |
| **AtBAP31** | At5g42570 | Sheep | 112-218 | V Good | Positive | 24.6 | OK |
| **BIG** | At3g02260 | Sheep | 4621-4820 | Good | Negative | 567.9 | >170, 75 |
| **BIM1** | At5g08130 | Sheep | 1-120 | Good | Positive | 45.5 | OK |
| **BIP1** | At5g28540 | Rabbit | 481-669 | Good | Positive | 73.6 | OK |
| **BRI1** | At4g39400 | Sheep | 1100-1196 | V Good | ? | 130.5 | ~140, 55, 43 |
| **Catalase2 (G)** | At4g35090 | Sheep | 1-492 | Good | Positive | 56.9 | OK |
| **AtCesA2** | At4g39350 | Sheep | 561-822 | V Good | Negative | 122.1 | ~67 |
| **AtCOB** | At5g60920 | Sheep | 256-430 | Good | ? | 51.2 | OK & a few faint bands |
| **γ-COP** | At4g34450 | Rabbit | 601-810 | Good | Positive | 98.5 | OK |
| **CTR1** | At5g03730 | Sheep | 1-210 | VVV Poor | Negative | 90.3 | ~40 |
| **EIL1** | At2g27050 | Sheep | 361-584 | Good | Positive(?) | 66.5 | 95 |
| **EIN2** | At5g03280 | Sheep | 599-998 | V Poor | ? | 141 | OK |
| **EIN3** | At3g20770 | Sheep | 421-628 | V Good | ? | 71.4 | No Band |
| **ETR1** | At1g66340 | Sheep | 584-738 | Poor | ? | 82.6 | No Band |
| **ETR1 (Generic)** | At1g66340 | Sheep | 115-343 | V Poor | ? | 82.6 | No Band |
| **GA2** | At1g79460 | Sheep | 331-500 | Good(?) | Positive(?) | 89.6 | No Band |
| **GA20ox** | At4g25420 | Sheep | 211-377 | V Poor | Positive(?) | 43.2 | No Band |
| **GA2ox2** | At1g30040 | Sheep | 1-341 | V Poor | ? | 38.5 | No Band |
| **GA3** | At5g25900 | Sheep | 241-450 | V Good | Positive | 58.2 | ~45, 50, 72, 130 |
| **GAI** | At1g14920 | Sheep | 1-145 | V Good | Negative | 58.9 | OK |
| **GNOM** | At1g13980 | Sheep | 934-1033 | Poor | Positive | 162.6 | OK |
| **KOB1** | At3g08550 | Sheep | 181-330 | V Good | Negative | 59.9 | 24 |
| **AtMDH** | At3g47520 | Rabbit | 261-390 | Good | Positive | 42.4 | OK |
| **NUC (generic)** | At5g44160 | Sheep | 37-196 | V Poor |  | 51.2 | ~120, 80 |
| **AtPHT3** | At5g14040 | Rabbit | 101-230 | V Poor |  | 40.1 | 95,55 |
| **PID** | At2g34650 | Sheep | 1-100 | Good | ? | 49.3 | No Band |
| **PIN1** | At1g73590 | Sheep | 155-408 | Not done | Positive | 67 | ~95, 120 and larger |
| **PIN1** | At1g73590 | rabbit | 155-408 | Not done | Positive | 67 | NT |
| **PIN2** | At5g57090 | Sheep | 315-479 | Not done | Positive | 69.3 | ~95 |
| **PIN2** | At5g57090 | rabbit | 315-479 | Not done | Positive | 69.3 | NT |
| **PIN3** | At1g70940 | Sheep | 289-470 | Not done | Positive | 69.5 | No Band |
| **PIN3** | At1g70940 | rabbit | 289-470 | Not done | Positive | 69.5 | NT |
| **PIN4** | At2g01420 | Sheep | 286-447 | Not done | Positive | 66.7 | ~84 |
| **PIN6** | At1g77110 | Sheep | 218-401 | V Poor | Positive | 62 | ~115, 30 |
| **PIN6** | At1g77110 | rabbit | 218-401 | Not done | Positive | 62 | NT |
| **PIN7** | At1g23080 | Sheep | 292-445 | V Good | Positive | 67.6 | ~62 |
| **PLT1g** | At3g20840 | Sheep | 175-327 | V Poor |  | 62.9 | ~120 |
| **PM-ATPase** | At2g18960 | Rabbit | 401-600 | Not done | Positive | 104.2 | OK |
| **QUA1** | At3g25140 | Sheep | 43-249 | V Poor | Negative | 64.4 | ~52 |
| **QUA2** | At1g78240 | Sheep | 106-272 | V Good | Negative | 77.9 | 38 |
| **RCN1** | At1g25490 | Sheep | 204-331 | V Good | ? | 65.5 | OK |
| **RGA** | At2g01570 | Sheep | 1-197 | V Good | ? | 64 | OK |
| **RHD1** | At1g64440 | Sheep | 121-348 | V Good | Negative | 38.1 | OK doublet, ~50 |
| **RSW1/CesA6/IRX2** | At5g64740 | Sheep | 391-644 | Good | Negative | 122.5 | OK? |
| **RSW2** | At5g49720 | Sheep | 352-566 | Poor | Negative | 69.2 | ~95 |
| **RSW3** | At5g63840 | Sheep | 734-921 | V Poor | Negative | 104.3 | OK |
| **SHR** | At4g37650 | Sheep | 10-103 | V Good | Positive | 59.5 | ~75 |
| **SLY1** | At4g24210 | Sheep | 1-151 | Good | Positive | 17.5 | >130, 95 |
| **SNX1** | At5g06140 | Sheep | 281-402 | Good(?) | ? | 46.5 | OK, faint |
| **SPY** | At3g11540 | Sheep | 451-640 | Poor | Negative | 101.4 | ~78 |
| **AtSYP21/AtPEP12** | At5g16830 | Rabbit | 6-210 | Poor |  | 31.1 | OK & ~150 faint |
| **AtSYP41TLGb** | At5g26980 | Rabbit | 81-240 | Poor |  | 36.1 | OK |
| **TIR1** | At3g62980 | Sheep | 359-499 | V Good | Negative | 66.8 | OK |
| **TWD** | At3g21640 | Sheep | 1-110 | V Good | Negative | 41.8 | OK |
| **Unknown protein** | At1g02330 | Sheep | 101-279 | Poor | ? | 32.4 | No Band |
| **Vacuolar Invertase** | At1g12240 | Sheep | 251-430 | Good | Positive | 73.8 | ~50, 30 |
| **VAN3** | At5g13300 | Sheep | 329-506 | Poor | Negative | 92.5 | No Band |
| **WAR1** | At2g31190 | Sheep | 291-433 | Poor | ? | 48.3 | No Band |
| **WXR3** | At3g45890 | Sheep | 421-608 | V Good | Positive | 66.4 | ~50, 45, 43 |

*Single correct size bands are indicated as ‘OK’. Where bands do not match the correct size or there are multiple bands, approximate band sizes (kDa) are indicated. NT- Not tested.
